# Processing Functional Near Infrared Spectroscopy Signal with a Kalman Filter to Assess Working Memory during Simulated Flight

**DOI:** 10.3389/fnhum.2015.00707

**Published:** 2016-01-19

**Authors:** Gautier Durantin, Sébastien Scannella, Thibault Gateau, Arnaud Delorme, Frédéric Dehais

**Affiliations:** ^1^Département Conception et Conduite des Véhicules Aéronautiques et Spatiaux, Institut Supérieur de l'Aéronautique et de l'Espace (ISAE-Supaéro)Toulouse, France; ^2^Centre de Recherche Cerveau et Cognition, Université Toulouse III - Paul SabatierToulouse, France; ^3^Centre National de la Recherche Scientifique, Centre de Recherche Cerveau et CognitionToulouse, France

**Keywords:** fNIRS, Kalman filtering, Neuroergonomics, working memory, SVM

## Abstract

Working memory (WM) is a key executive function for operating aircraft, especially when pilots have to recall series of air traffic control instructions. There is a need to implement tools to monitor WM as its limitation may jeopardize flight safety. An innovative way to address this issue is to adopt a Neuroergonomics approach that merges knowledge and methods from Human Factors, System Engineering, and Neuroscience. A challenge of great importance for Neuroergonomics is to implement efficient brain imaging techniques to measure the brain at work and to design Brain Computer Interfaces (BCI). We used functional near infrared spectroscopy as it has been already successfully tested to measure WM capacity in complex environment with air traffic controllers (ATC), pilots, or unmanned vehicle operators. However, the extraction of relevant features from the raw signal in ecological environment is still a critical issue due to the complexity of implementing real-time signal processing techniques without a priori knowledge. We proposed to implement the Kalman filtering approach, a signal processing technique that is efficient when the dynamics of the signal can be modeled. We based our approach on the Boynton model of hemodynamic response. We conducted a first experiment with nine participants involving a basic WM task to estimate the noise covariances of the Kalman filter. We then conducted a more ecological experiment in our flight simulator with 18 pilots who interacted with ATC instructions (two levels of difficulty). The data was processed with the same Kalman filter settings implemented in the first experiment. This filter was benchmarked with a classical pass-band IIR filter and a Moving Average Convergence Divergence (MACD) filter. Statistical analysis revealed that the Kalman filter was the most efficient to separate the two levels of load, by increasing the observed effect size in prefrontal areas involved in WM. In addition, the use of a Kalman filter increased the performance of the classification of WM levels based on brain signal. The results suggest that Kalman filter is a suitable approach for real-time improvement of near infrared spectroscopy signal in ecological situations and the development of BCI.

## 1. Introduction

The development of passive Brain Computer Interfaces (BCI) is a key topic of research in Neuroergonomics. In contrast with active ones, Passive BCI (Cutrell and Tan, 2008) allows the use of unintentionally produced brain activity to derive various cognitive states (Blankertz et al., 2010) such as excessive mental workload. Such states inference provides an interesting insight as they aim at dynamically adapting the nature of the human-system interactions to overcome cognitive limitations (Zander and Kothe, 2011; Brouwer et al., 2013). In the field of BCI design to enhance user performance, there is a growing interest for functional near infrared spectroscopy (fNIRS) based BCI (Coyle et al., 2004; Derosière et al., 2014; Strait et al., 2014). This brain imaging device uses near infrared light absorption properties to estimate local variations of cortical hemodynamics. It uses a modified Beer-Lambert law to link light transmittance through brain tissues to variations in local concentrations in oxygenated hemoglobin (*HbO*_2_) and deoxygenated hemoglobin (*HHb*) (Villringer and Obrig, 2002). fNIRS has a good spatial resolution (around 1 cm^2^) and interesting signal-to-noise ratio. Moreover, this technique has the advantage to be easy and fast to set over the participant's head with a short calibration process (Naseer and Hong, 2015). However, the processing of fNIRS signal faces a lack of methodological consensus and thus still represents a great challenge (Bashashati et al., 2007). The extraction of the relevant activity from brain signals requires complex techniques (van Erp et al., 2012), and most efficient ones often rely on long calibration times [e.g., in subspace artifact removal techniques (von Bünau et al., 2009), adaptive filtering (Zheng et al., 2002)]. The complexity of these methods limits their applicability for Neuroergonomics purpose, as the signal has to be useable in real-time.

Most BCI designs rely on classical linear bandpass filtering techniques such as Infinite Impulse Response (IIR) (Naseer and Hong, 2015), although current research focuses on the investigation of alternative signal processing techniques, such as the Moving Average Convergence Divergence (MACD) filter (Durantin et al., 2014b; Gateau et al., 2015). On this basis, the improvement of signal quality in real-world conditions as suggested in the Neuroergonomics approach, makes Kalman filtering an ideal candidate. This signal processing and estimation technique relies both on the measurements performed on a system and on a modeling of its dynamics to improve signal quality (Kalman, 1960). The use of a Kalman filter including a physiological model of brain function to improve signal usability has been previously applied to EEG (Georgiadis et al., 2005; Callan et al., 2015) or fMRI (Diamond et al., 2005). However, concerning fNIRS, this technique has been limited to the estimation of model parameters (Abdelnour and Huppert, 2009) or the correction of motion artifacts (Izzetoglu et al., 2010), therefore not requiring the use of a physiological model of hemodynamic response to stimulation.

One of the greatest challenges regarding Kalman filter design is the tuning of its parameters, i.e., to evaluate the level of measurement noise (*R*) affecting the signal and the state noise (*Q*) in the model (Diamond et al., 2005). The value of the ratio *Q*∕*R* greatly influences the behavior of the Kalman filter. Indeed, a Kalman filter with a low value of *Q*∕*R* will put confidence in the dynamical model, whereas a Kalman filter with a high value of *Q*∕*R* will put confidence in the measurements. In practice, the value of this ratio often has to be chosen empirically (Abdelnour and Huppert, 2009; Callan et al., 2015), as there exists no efficient way to evaluate it. Consequently, the dynamics of the Kalman filter may not be adapted to the data needed to be improved. The challenge of this study was to design a Kalman filter suitable for fNIRS that includes a physiological model of hemodynamic response (Boynton et al., 1996). By applying this filter to fNIRS data collected during both controlled and ecological experiments, we also aimed at testing the improvements such a filter could bring to fNIRS signal toward the implementation of a passive BCI. To that end, we first designed a Kalman filter relying on a model of the hemodynamic response (Boynton et al., 1996) to improve signal quality. We then conducted a first experiment with a prefrontal fNIRS, involving a digit sequence memorization task used to measure Working Memory (WM) storage and update capacity. Provided that the development of a signal improvement technique usable in realistic operational settings was the objective of this study, this basic task was chosen as WM is a key executive function to operate complex systems (Causse et al., 2011). Data collected during the first experiment were used to select the value of the filter parameter *Q*∕*R* using an optimization procedure. Finally, the improvement of the signal by the optimal Kalman filter was evaluated with formal classification during an ecological experiment which involved pilots performing a realistic WM task (i.e., recalling air traffic instructions) in a flight simulator.

## 2. Kalman filter design

The functional model used to design the Kalman filter for fNIRS signal was inspired by the Hemodynamic Response Function (HRF) proposed by Boynton et al. (1996). This function is simple enough to be represented by a low order state-space model. This model assumes a third order impulsional response to stimulation, and has the following transfer function :

(1) $$\begin{array}{l} HRF\left(p\right)=\frac{\tau^{3}e^{-\delta p}}{{\left(p+\tau \right)}^{3}} \end{array}$$

**Figure 1:**
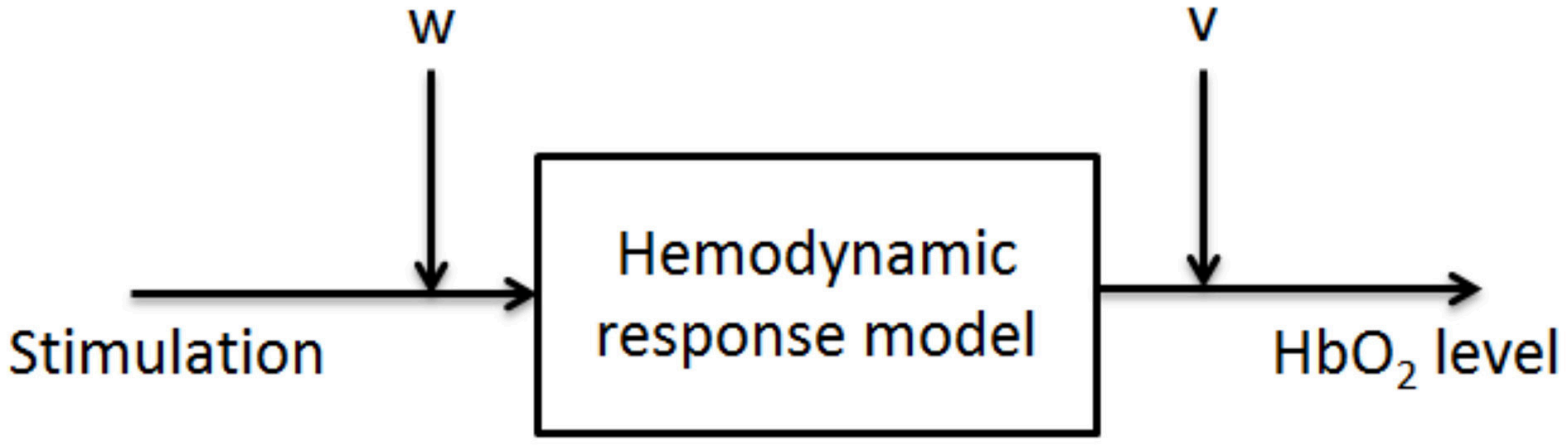
**Proposed approach for Kalman filtering of fNIRS**. The state noise is considered as a perturbation of the input of the system.

As shown in Equation (1), the response shape depends on two parameters : δ represents the pure delay between stimulation and the start of *HbO*_2_ increase ; τ influences the time-to-peak delay. Typical values that were chosen here were extracted from Boynton et al. (1996), and are δ = 2*s* and τ = 1.5*s*. This choice leads to a time-to-peak delay from pulse stimulation of around 5 *s* (Handwerker et al., 2004). Then, the Kalman filter principle requires the addition to the model of a state noise *w* (defined as the amount of noise affecting the model, i.e., the amount of errors in it) and of a measurement noise *v* (defined as the amount of noise affecting the measures). As shown on Figure 1, we chose to represent the state noise as a perturbation affecting the stimulus (i.e., the input of the model). This choice led us to consider that state noise represents a stimulus perception (or internalization) bias.

The perception bias perturbing the stimulus is noted *b*. In the nominal model, Kalman filter assumptions impose that *b* = *w*, where *w* is the state noise following a gaussian centered distribution. This model, in addition to the choice of a *Q*∕*R* value (where *Q* is the variance of the state noise, and *R* is the variance of the measurement noise), allowed us to design a *Kalman filter* for fNIRS signal improvement. The inputs of the Kalman filter were the stimuli onsets and the fNIRS raw signal.

One of the main limitations of this approach is the fact that the stimulus perception bias has to be centered (i.e., *b* = 0 on average), which can be erroneous when the subject sustainably disengages from the task and doesn't pay attention to the stimuli. To take this element into account, we built a second model, which is an augmentation of the nominal model, and in which ḃ = *w*. Thus, as the first derivative of *b* follows the gaussian centered distribution, it is still possible to design a Kalman filter, without assuming that *b* is null on average. This augmented model, along with the value of *Q*∕*R*, allowed the computation of the *augmented Kalman filter* for fNIRS signal processing. For both filters, the value of the ratio *Q*∕*R* was fixed according to an optimization process (see next section).

## 3. First step : Setting the filter parameters

### 3.1. Material and methods

**Figure 2:**
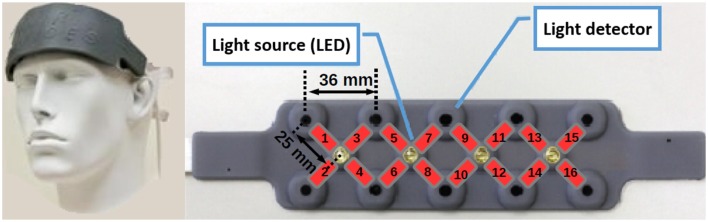
**fNIRS device optodes location**. The device is composed of four light sources and 10 light detectors. The association of one light source and one light detector composes the optodes. The disposition of the sources and detectors leads to 16 optodes over the prefrontal cortex.

Nine healthy participants from the Institut Superieur de l'Aeronautique et de l'Espace (ISAE ; Mean age = 21.6; *SD* = 1.5; eight males, eight right handed) participated in the experiment. The volunteers performed a computer-based digit sequence memorization task, while fNIRS measurements of the prefrontal cortex were recorded. Data were recorded using a Biopac® fNIR100 device, composed of 16 optodes placed on the forehead (see Figure 2). Each optode of the device records hemodynamics at a frequency of 2 Hz in term of oxygenated hemoglobin (*HbO*_2_) and deoxygenated hemoglobin (*HHb*) level variations in comparison to a baseline.

**Figure 3:**
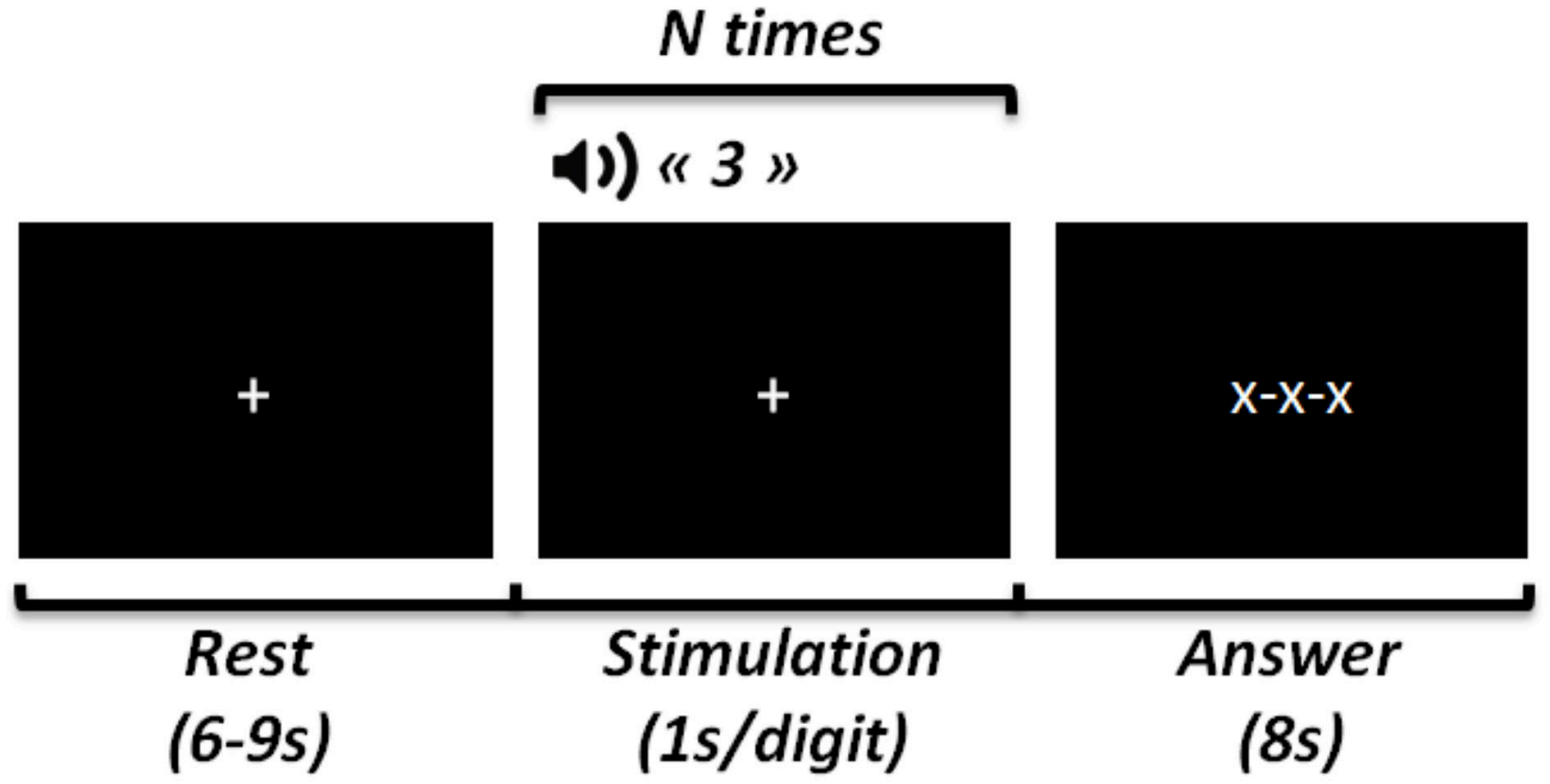
**Time course of one trial of the experiment**. The experiment consisted of a total of 27 trials (three for each level of difficulty), presented in a random order. *N* = digit sequence size (5, 7, or 9).

Each trial of the experiment consisted in the memorization of a sequence of 5, 7, or 9 randomly chosen digits. The size of the sequence defined a level of difficulty. Figure 3 summarizes the time sequence of a trial. During each trial, the subjects were asked to look at a fixation cross at the center of the screen. The digit sequence was presented through the loudspeakers of the computer using prerecorded audio tracks, at a rate of one digit per second. After the presentation of the last digit, the fixation cross was replaced by three crosses, indicating that the subjects had 8 *s* to type the memorized sequence on the keyboard. Between two consecutive trials, the subjects looked passively at the fixation cross at the center of the screen for 6 to 9 *s* (the inter-trial interval was chosen randomly to avoid task periodicity). The experiment consisted of 27 trials (nine trials for each of the three levels of difficulty), presented in a randomized order.

### 3.2. Data processing

Data were processed using Matlab®. Two different types of Kalman filters were applied to the data, the *nominal Kalman filter* (in which we assumed that the stimulus perception bias is null on average) and the *augmented Kalman filter* (without this assumption). The inputs for both filters were the stimuli onsets and the raw fNIRS data. For each filter, the value of the *Q*∕*R* ratio chosen for the Kalman filter tuning ranged from 10^−5^ to 10^5^, in order to look for the optimal results. Simultaneously, we also applied the MACD filter (Durantin et al., 2014b) to raw data in order to compare Kalman results with classical filtering.

For each trial, we computed the *HbO*_2_ peak response (noted Δ*HbO*_2_), i.e., the difference between the maximum value of *HbO*_2_ in the 30 s following the trial onset and the value of *HbO*_2_ at onset time. We similarly computed the *HHb* peak response (noted Δ*HHb*).

**Figure 4:**
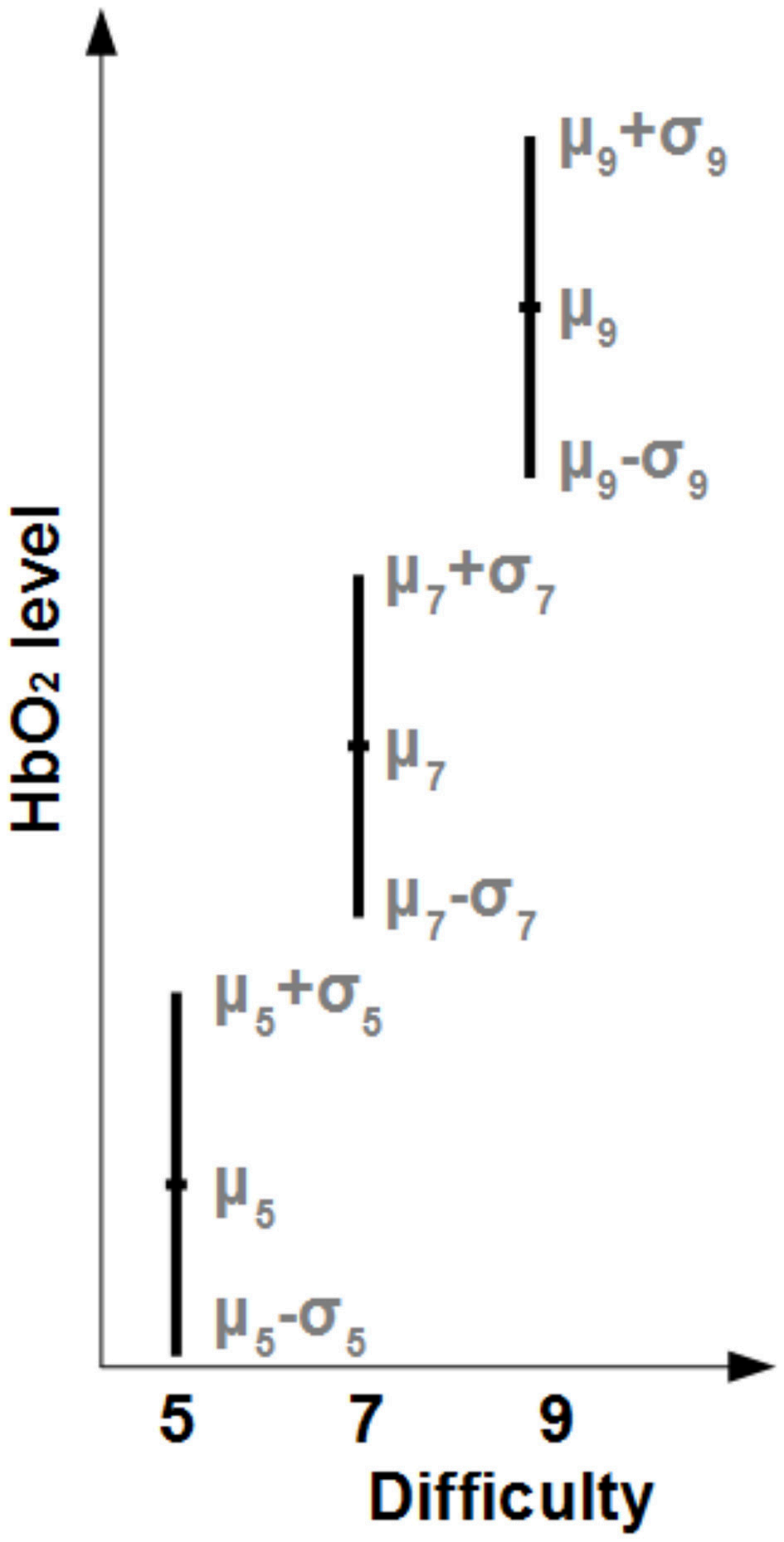
**Illustration of the computation of the Effect Size Index (ESI) for ***HbO***_**2**_**. We computed the mean (μ) and standard deviation (σ) of the level of *HbO*_2_ across subjects for each difficulty. For a difficulty *N*, a confidence interval corresponding to one standard deviation was computed as [μ_*N*_ − σ_*N*_; μ_*N*_ + σ_*N*_]. The corresponding ESI was computed as the sum of the gaps between the confidence intervals (negative if the confidence intervals are overlapping).

Preliminary, the potential good values for *Q*∕*R* (i.e., those leading to improvement in the signal) were isolated by computing an *Effect Size Index* (ESI, illustrated on Figure 4. For each filter (MACD, nominal Kalman or augmented Kalman with a given *Q*∕*R* value) and each digit sequence size *N*, we computed the mean (μ_*N*_) and standard deviation (σ_*N*_) of the level of Δ*HbO*_2_ or Δ*HHb* measured at each optode. We used these values to compute a confidence interval corresponding to one standard deviation as [μ_*N*_ − σ_*N*_; μ_*N*_ + σ_*N*_]. The *ESI* was defined as the gap between the confidence intervals of each condition (negative if the confidence intervals are overlapping), i.e.,

$$\begin{array}{l} ESI=\left(\left(\mu_{7}-\sigma_{7}\right)-\left(\mu_{5}+\sigma_{5}\right)\right)+\left(\left(\mu_{9}-\sigma_{9}\right)-\left(\mu_{7}+\sigma_{7}\right)\right) \end{array}$$

We then proceeded to visual inspection to find the best values for *Q*∕*R* ratio, by finding the parameters leading to higher ESI values. Each set of data was finally tested using a Two-way analysis of variance ANOVA, with two factors (16 optodes, three levels of difficulty), performed using STATISTICA® software. The strength of the statistical effect of the difficulty level, evaluated using the partial η^2^, was used to compare the results of the different filters.

### 3.3. Results

**Figure 5:**
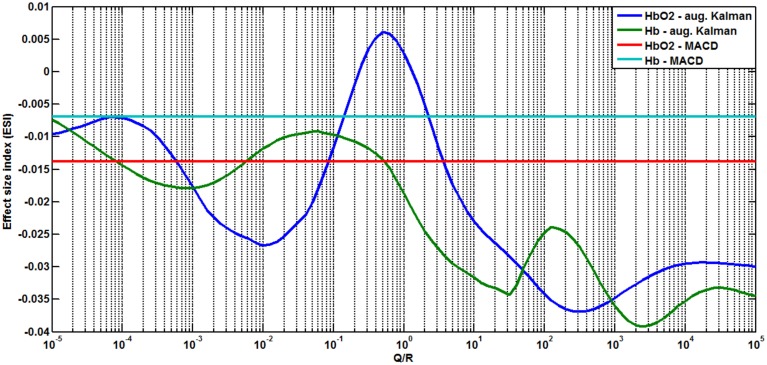
**Estimated Effect Size Index (ESI) for the augmented Kalman filter (for ***HHb*** –in green– and ***HbO***_**2**_ –in dark blue–) in function of the value of ***Q***∕***R***, compared to the ESI of the MACD filter (in red for ***HbO***_**2**_, in light blue for ***HHb***)**. The index is estimated using the data from optode 2.

As shown on Figure 5, the optimal results were obtained for *HbO*_2_ at optode 2 recording mainly from the left inferior frontal gyrus, when using the nominal Kalman filter with *Q*∕*R* = 3.98 or the augmented Kalman filter with *Q*∕*R* = 0.50. Table 1 summarizes the effect sizes obtained for each of the signal processing techniques tested (those effects showed an increase in the level of Δ*HbO*_2_ with growing sequence sizes).

**Table 1:** **Effect sizes obtained for the effect of difficulty over all the subjects for the level of Δ***HbO***_**2**_ measured at optode 2, depending on the type of filter used for signal processing**.

**Filter type**	***Q*∕*R* ratio**	**partial η^2^**
MACD		0.21
Nominal Kalman	3.98	0.32
Augmented Kalman	0.50	0.34

**Figure 6:**
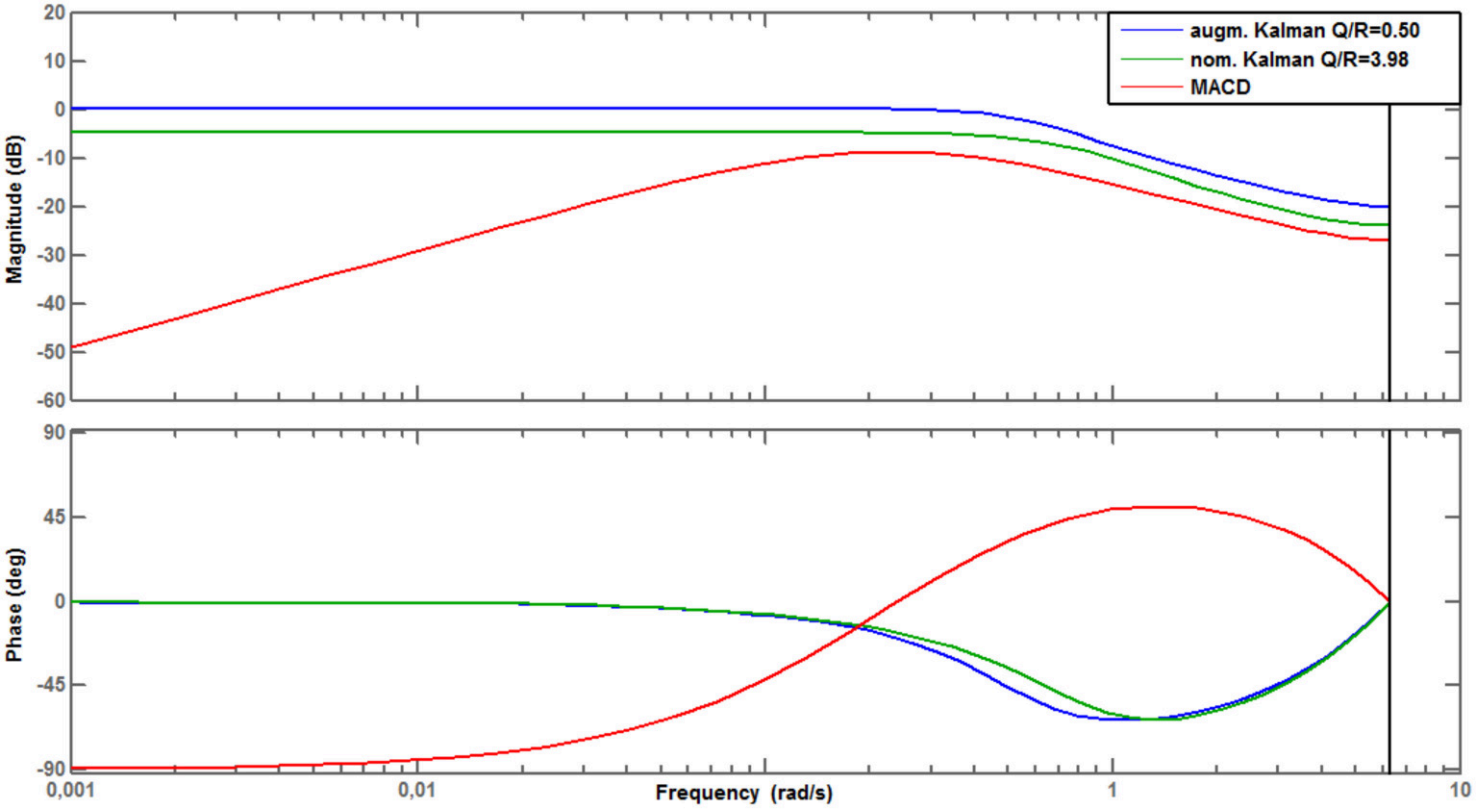
**Bode diagrams (frequency response, phase response) of the MACD filter, the nominal, and augmented Kalman filters**.

The frequency and phase responses (Bode diagram) of the nominal Kalman filter (*Q*∕*R* = 3.98) and of the augmented Kalman filter (*Q*∕*R* = 0.50) are given on Figure 6. As the two filters exhibit similar Bode diagrams (and therefore similar filtering properties), we retained only the augmented Kalman filter for testing on new data.

## 4. Second step : Testing the applicability of the filter in ecological conditions

### 4.1. Material and methods

**Figure 7:**
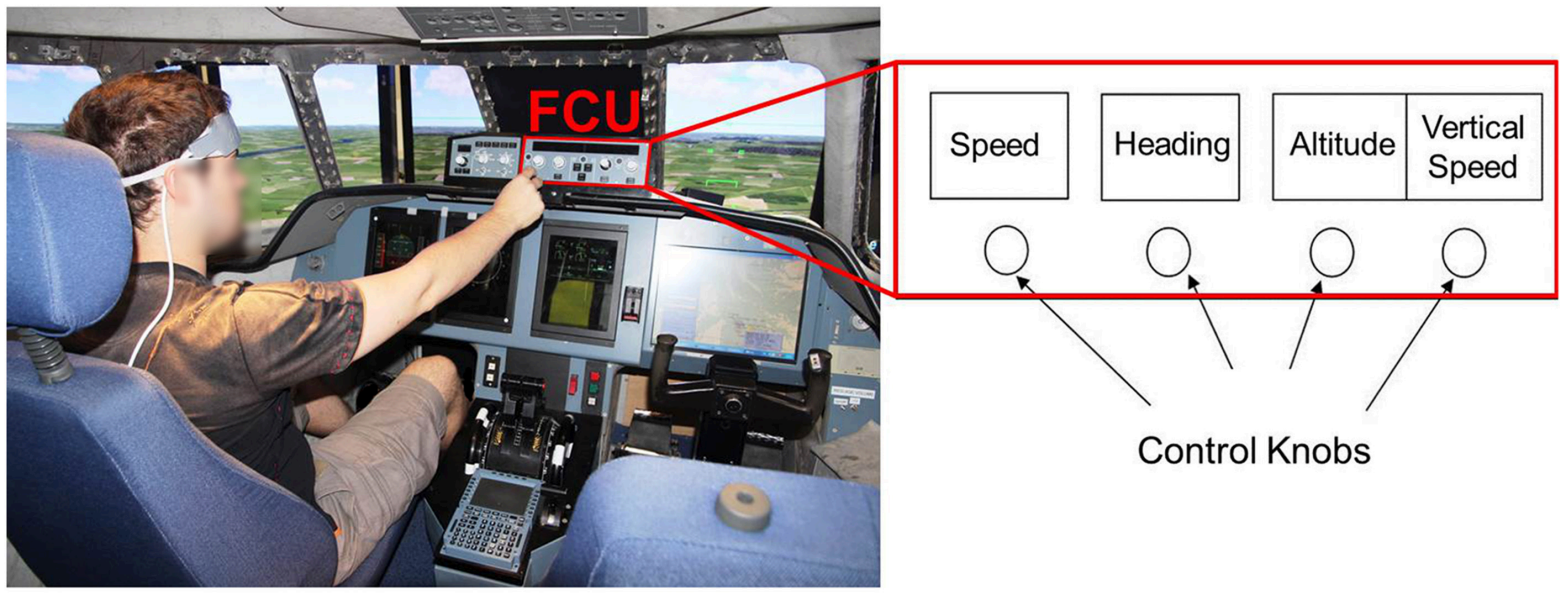
**Pilot's interaction with the auto flight system**. The participants controlled the aircraft simulator of ISAE from the left seat. The red rectangle corresponds to the FCU (Flight Control Unit) dedicated to set the autopilot using the four control knobs, accordingly to ATC (Air Traffic Control) clearances (speed, heading, altitude, and vertical speed selection). Adapted from Gateau et al. (2015).

Data used for testing the Kalman filter were extracted from a second experiment involving a digit sequence memorization in a realistic flight simulator (see Figure 7 for an illustration of the setup). The experiment was similar to Gateau et al. (2015), and included 18 healthy subjects (Mean age = 27.1; *SD* = 6.4; six women). Pilots heard prerecorded Air Traffic Controller (ATC) messages and were asked to dial the corresponding flight parameters in the Flight Control Unit (FCU) using the four knobs (i.e., speed, heading, altitude, and vertical speed knobs) of the FCU. The ATC messages were delivered at 78 dB SPL trough a Sennheiser® headset. We defined two levels of difficulty depending on the complexity of the message:

- Low Load trial difficulty: only one major value per trial was used to set each flight parameter (e.g., 15 for “speed 150, heading 150, altitude 1500, vertical speed +1500”).
- High Load trial difficulty: each flight parameter value was different from the previous one and composed of different digits to maximize the complexity (e.g., “speed 164, heading 235, altitude 8700, vertical speed −1600”).

The task consisted in 20 repetitions of each difficulty for a total of 40 trials. Each ATC message started with the airplane call sign (i.e., “Supaero 32”), followed by the sequence of flight parameters. It ended with the message “over.” The subjects were instructed to set the parameters strictly only after they heard the “over” message. A practice session was conducted for each subject before the actual experiments to allow them to become familiar with the experiment and the interface. After each message, the pilots had 18 seconds to enter the flight parameters. Trials were separated by 11 to 13 s of rest. During the experiment, hemodynamics of the prefrontal cortex were recorded using the same device than in the first experiment.

### 4.2. Data processing and classification

The raw *HbO*_2_ data measured at each optode were filtered using three types of filter. First, we used the MACD filter and the augmented Kalman filter retained from the optimization phase (*Q*∕*R* = 0.50). We also used a classical IIR Butterworth bandpass filter (0.02 Hz < *f* < 0.1 Hz), in order to compare the results to classical filtering. The statistical effect sizes of the level of Δ*HbO*_2_ (computed in the same way than in the first experiment) were evaluated using repeated measures ANOVA performed with STATISTICA®. The performance of the different filters were compared in terms of partial η^2^. In addition, we computed the statistical *t*-maps representing the differences in the contrasts between high and low load conditions in terms of level of *HbO*_2_ for each type of filters. This computation was done using Matlab and plotted using the topograph tool from fNIRSoft®.

The improvement of the signal depending on the type of filter used for processing was also evaluated by performing formal classification on the data. This analysis was performed using the Statistics and Machine Learning toolbox from Matlab. The Δ*HbO*_2_ values extracted from each optode were used to train and test a Linear Support Vector Machine (SVM) classifier through a 10-fold cross validation process : for each subject, data from all trials were randomly divided in 10. The difficulty (high or low load) of the trials of each 10% of data was predicted by a SVM classifier that was previously trained on the 90% remaining data. The predicted labels were then examined to evaluate the *Accuracy* (probability of good classification), *Sensitivity* (probability of good classification for high load trials), and *Specificity* (probability of good classification for low load trials) of the classifier.

### 4.3. Results

The partial η^2^ obtained for each type of filters are given in Table 2 (for each optode and across all the optodes). The results show that the use of MACD elicits a better statistical effect size than the classical IIR filter. Similarly, the use of Kalman filter yields better results than both MACD and IIR filters. This result is true not only when filtering data from optode 2, but present notably at all optodes located in the bilateral dorsolateral areas of the prefrontal cortex (optodes 1, 2, 3, 4 and 13, 14, 15, 16).

**Table 2:** **Effect sizes (partial η^**2**^) obtained for the effect of difficulty over all the subjects for the level of ***HbO***_**2**_ measured for each optodes (plus main effect size over all optodes), depending on the type of filter used for signal processing**.

**Optode number**	**1**	**2**	**3**	**4**	**5**	**6**	**7**	**8**	
**IIR**	0.34	0.36	**0.48**	0.35	0.15	0.32	0.32	0.00	
**MACD**	**0.51**	**0.44**	**0.50**	**0.53**	0.24	0.26	0.15	0.01	
**Augmented Kalman** (*Q*∕*R* = 0.50)	**0.64**	**0.55**	**0.57**	**0.55**	0.38	0.36	0.39	0.15	
**Optode number**	**9**	**10**	**11**	**12**	**13**	**14**	**15**	**16**	**All**
**IIR**	0.29	0.26	0.32	0.11	**0.41**	0.34	0.33	0.30	0.36
**MACD**	0.15	0.01	0.31	0.12	**0.50**	0.25	0.39	0.38	0.33
**Augmented Kalman** (*Q*∕*R* = 0.50)	**0.42**	0.35	**0.53**	0.39	**0.55**	**0.55**	**0.52**	**0.49**	**0.52**

*The effect sizes corresponding to a significant effect (p < 0.05) after correction for multiple comparisons are reported in bold font*.

**Figure 8:**
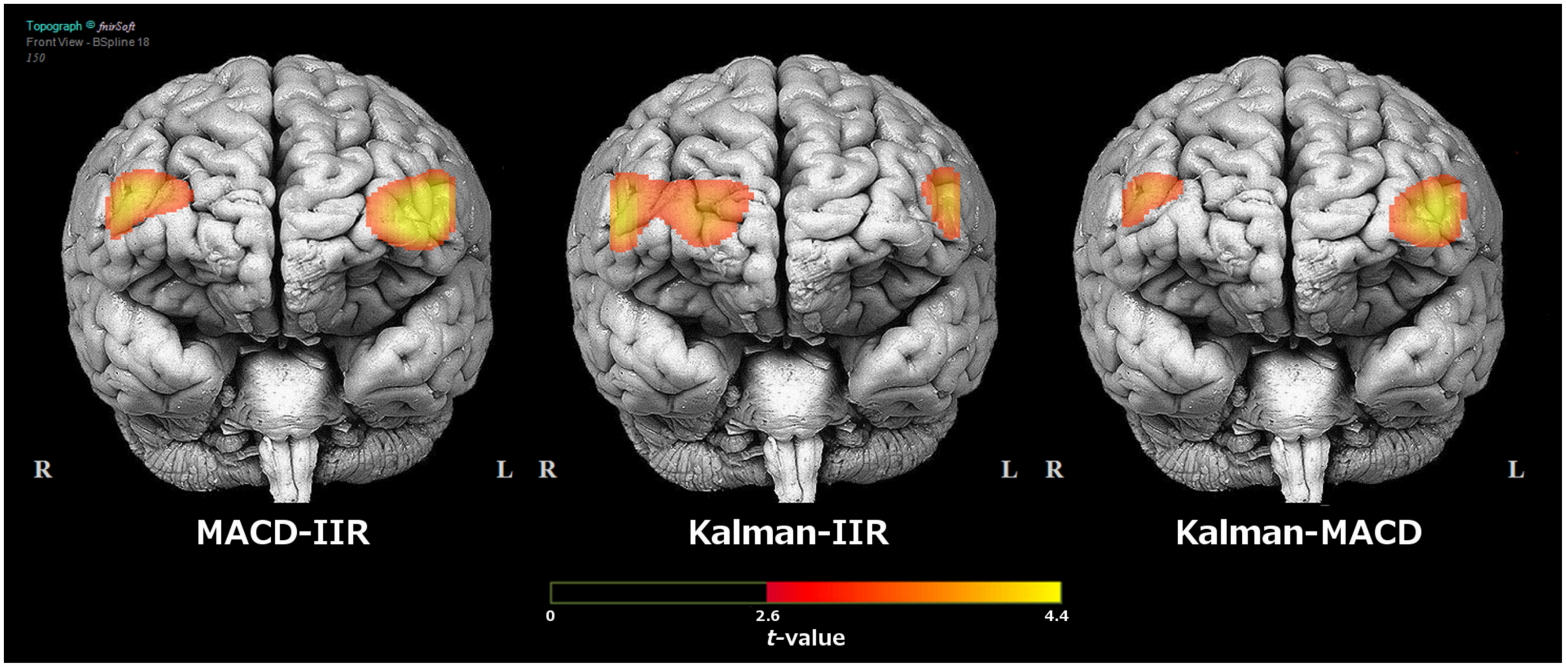
**Comparison of the ***t***-maps for the contrast ***High load - Low load*** on the level of ***HbO***_**2**_ over the prefrontal cortex obtained in function of the type of filter used for signal processing (classical IIR, MACD, or Kalman)**. The topographical view was extracted from fNIRSoft® and the threshold was fixed at the statistical significance level with α = 0.01, to account for multiple comparisons.

The effect of trial difficulty on the level of *HbO*_2_ measured over the prefrontal cortex is shown on Figure 8. On this figure, we observe that both the MACD and Kalman filter over classical IIR filter improve the discriminability between the two conditions in the lateral areas of the prefrontal cortex.

**Figure 9:**
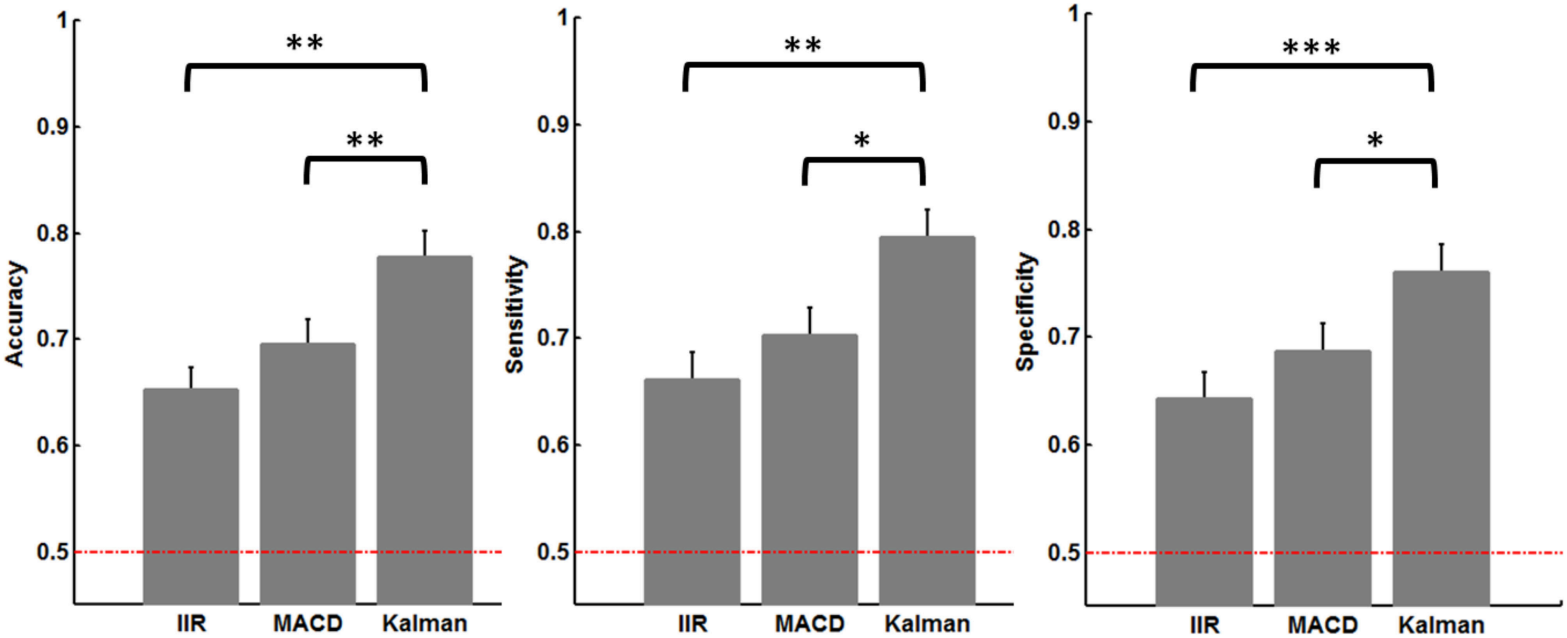
**Accuracy, sensitivity, and specificity obtained from the 10-fold cross-validation procedure for each type of filter**. The dashed red line represents chance level (50%). The error bars represent the standard error of the mean across subjects, and statistical significance after correction for multiple comparisons is indicated by stars (^*^p < 0.05 ; ^**^p < 0.01 ; ^***^p < 0.001).

Ultimately, the cross-validation procedure performed on the data to classify low-load vs. high-load trials are presented in Figure 9 in terms of accuracy, sensitivity, and specificity. The classification results were all significantly better than chance, although Kalman filter led to statistically better results than IIR and MACD filters. Using Kalman filtering, the classification accuracy reached 77.8%, with a sensitivity of 79.4% and a specificity of 76%.

## 5. Discussion

The objective of the study was to design a Kalman filter to improve fNIRS signal for Neuroergonomics applications. In particular, the main challenge concerned the tuning of the parameters *Q* and *R* (Diamond et al., 2005), representing the state noise and measurement noise variances. Based on a simple model of the hemodynamic response to neuronal stimulation (Boynton et al., 1996), we designed a Kalman filter model taking into account both the measurement noise and the stimulus perception bias that can occur in periods of disengagement or when the level of attention varies. During an optimization process, we showed that it was possible to find values for the parameters which leads to better statistical results (*Q*∕*R* = 0.50) with an augmented model. Interestingly, the relatively low value of the *Q*∕*R* ratio in the second model suggests that the Kalman filter put more confidence in the dynamical model of hemodynamics response than in fNIRS data. The higher optimal value obtained for this ratio when using the first choice of model (*Q*∕*R* = 3.98) suggests this model was less consistent with the actual hemodynamics characteristics.

We applied the optimal results found in the first experiment on new data from an ecological experiment in a flight simulator, and showed that the optimal Kalman filter tuning could be applied generically. This filter led to higher effect sizes when looking at the effect of task difficulty in both tasks, compared to classical filters (see Figure 8). It is argued that the use of a dynamical physiological model by the Kalman filter implies less variability across trials and subjects, therefore explaining the greater stability of the results obtained with this filter. These results suggest that this filter would be suitable to improve the discriminability between the two conditions toward the implementation of a BCI to assist the operator, and would support the use of Kalman filtering to improve fNIRS signal (Izzetoglu et al., 2010). In particular, the Kalman filter helped us perform better during the SVM-based classification procedure between low-load and high-load trials, which confirms its contribution to the improvement of the signal. In addition, the experiment also confirmed that the MACD filter brings good results compared to classical IIR filtering, as it was previously demonstrated (Durantin et al., 2014b; Gateau et al., 2015). Although the discriminability obtained with this filter is not as good as the one obtained with the Kalman filter, it presents the advantage of not requiring any information on the stimulus onsets.

Interestingly, the optimal results for the first experiment were found at optode 2 recording mainly from the left inferior frontal gyrus. More generally, when applying the optimal Kalman filter in the second experiment, the WM solicitation elicited an activation of bilateral areas in the inferior and middle frontal gyri, part of the dorsolateral prefrontal cortex (see Figure 8 and Table 2). This result is in agreement with previous fNIRS studies that have found these regions are sensitive to WM solicitation (Ayaz et al., 2012; Durantin et al., 2014a). Therefore, the improvement of the fNIRS signal collected in this region suggests that this filter could be applied to any experiment recruiting the same functional areas. In particular, the optimization process carried in this study would avoid the need of a calibration phase or of a convergence phase (in case of adaptive filtering) to improve signal quality. However, further investigation is still needed to assess whether this filter could be used with the same model and tuning in experiments recruiting different brain areas. Similarly, further investigation is also needed to assess the usability of these filters in ecological conditions that would differ from a simulated flight (e.g., with higher levels of light variations or motion artifacts).

Nevertheless, some modifications of the model could lead to better usability and performance of the Kalman filter. For instance, the use of a stimulus onset detection technique such as the detection technique based on the MACD filter (Durantin et al., 2014b; Gateau et al., 2015) could replace the stimulus onsets input of the Kalman filter, therefore reducing the complexity of the filter. In addition, it would be interesting to compare the results of the current Kalman model relying on a simple modeling of the hemodynamic response to more complex physiological models (e.g., Buxton et al., 2004). Finally, using an adaptive *Q*∕*R* gain or realizing an optimization process for each subject instead of using a generic filter could also yield better results, although it would add complexity and a calibration phase to the procedure.

Altogether, the promising results of the study stand in favor of the use of Kalman filtering as a signal improvement technique for fNIRS signals with applications in Neuroergonomics. In particular, the improved signal would be available in real-time and without a calibration phase, and would allow better classification of WM levels in ecological settings.

## Author contributions

All the authors contributed to the experiment design, results discussion, and paper redaction. Data collection was made by GD, TG, and SS. Signal processing tools (Kalman filter, MACD), were developed by GD.

### Conflict of interest statement

The authors declare that the research was conducted in the absence of any commercial or financial relationships that could be construed as a potential conflict of interest.
